# Hydrogel forming microneedles loaded with VEGF and Ritlecitinib/polyhydroxyalkanoates nanoparticles for mini-invasive androgenetic alopecia treatment

**DOI:** 10.1016/j.bioactmat.2024.04.020

**Published:** 2024-04-23

**Authors:** Yan-Wen Ding, Yang Li, Zhi-Wei Zhang, Jin-Wei Dao, Dai-Xu Wei

**Affiliations:** a Key Laboratory of Resource Biology and Biotechnology in Western China, Ministry of Education, School of Medicine, Department of Life Sciences and Medicine, Northwest University, Xi'an, 710069, China; b School of Clinical Medicine, Chengdu University, Chengdu, China; c Dehong Biomedical Engineering Research Center, Dehong Teachers' College, Dehong, Yunnan Province, China; d Shaanxi Key Laboratory for Carbon Neutral Technology, Xi'an, 710069, China

**Keywords:** Hydrogel forming microneedles, Polyhydroxyalkanoates, VEGF, Ritlecitinib, Androgenetic alopecia treatment

## Abstract

Androgenetic alopecia (AGA), the most prevalent clinical hair loss, lacks safe and effective treatments due to downregulated angiogenic genes and insufficient vascularization in the perifollicular microenvironment of the bald scalp in AGA patients. In this study, a hyaluronic acid (HA) based hydrogel-formed microneedle (MN) was designed, referred to as V-R-MNs, which was simultaneously loaded with vascular endothelial growth factor (VEGF) and the novel hair loss drug Ritlecitinib, the latter is encapsulated in slowly biodegradable polyhydroxyalkanoates (PHAs) nanoparticles (R-PHA NPs) for minimally invasive AGA treatment. The integration of HA based hydrogel alongside PHA nanoparticles significantly bolstered the mechanical characteristics of microneedles and enhanced skin penetration efficiency. Due to the biosafety, mechanical strength, and controlled degradation properties of HA hydrogel formed microneedles, V-R-MNs can effectively penetrate the skin's stratum corneum, facilitating the direct delivery of VEGF and Ritlecitinib in a minimally invasive, painless and long-term sustained release manner. V-R-MNs not only promoted angiogenesis and improve the immune microenvironment around the hair follicle to promote the proliferation and development of hair follicle cells, but also the application of MNs to the skin to produce certain mechanical stimulation could also promote angiogenesis. In comparison to the clinical drug minoxidil for AGA treatment, the hair regeneration effect of V-R-MN in AGA model mice is characterized by a rapid onset of the anagen phase, improved hair quality, and greater coverage. This introduces a new, clinically safer, and more efficient strategy for AGA treatment, and serving as a reference for the treatment of other related diseases.

## Highlights

• Designed a HA-hydrogel microneedles, simultaneously integrated VEGF and Ritlecitinib contained in PHA NPs for AGA treatment.
• R-PHA NP addition enhances microneedle hardness, aiding minimally invasive transdermal delivery of microneedles.
• V-R-MN group showed faster, superior hair regeneration than monotherapy (V-MN, R-MN, Ritlecitinib, and Minoxidil).
• V-R-MN shows high biocompatibility with skin, ensuring safety and feasibility for transdermal drug delivery.

## Graphical abstract

A hyaluronic acid (HA) based hydrogel-formed microneedle (MN) was designed, referred to as V-R-MNs, which was simultaneously loaded with vascular endothelial growth factor (VEGF) and the novel hair loss drug Ritlecitinib, the latter is encapsulated in slowly biodegradable polyhydroxyalkanoates (PHAs) nanoparticles (R-PHA NPs) for minimally invasive Androgenetic alopecia (AGA) treatment. The V-R-MNs promote angiogenesis and improve the immune microenvironment around the hair follicle, fostering the proliferation and development of hair follicle cells. In comparison to the clinical drug minoxidil for AGA treatment, V-R-MN exhibits a hair regeneration effect in AGA model mice characterized by a rapid onset of the anagen phase, improved hair quality, and greater coverage.

**Figure:**
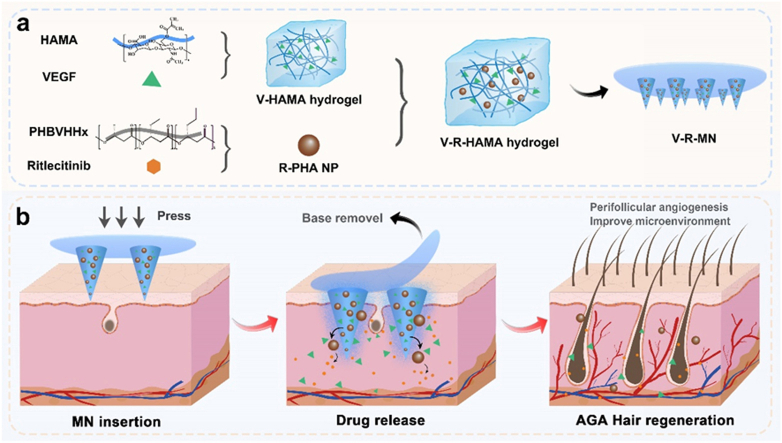

## 1 Introduction

Hair loss is a global challenge that causes adverse effects on patients such as obsessive-compulsive disorder, interpersonal, sensitivity, depression, fear, anxiety [1,2]. The majority of hair loss patients are due to Androgenetic alopecia (AGA) [3,4], primarily caused by dysregulation in the microenvironmental niche surrounding hair follicles, including vascular insufficiency or oxidative stress [[5], [6], [7]]. Nutrients essential for hair growth are supplied by blood vessels around hair follicles, delivering nutrients, cytokines, and other bioactive molecules [[8], [9], [10]]. The hair follicle growth cycle involves anagen, catagen, and telogen phases. Insufficient vascularization in the microenvironment around the hair follicle cannot meet the nutritional needs of hair follicle growth, resulting in shrinkage of the hair follicle and shortening of the growth phase, and which inhibits the transition of hair follicles from the telogen phase to the anagen phase, manifested as the gradual thinning of hair on the scalp [11,12]. While AGA is typically categorized as a noninflammatory and nonscarring form of alopecia, histological evidence of inflammation has been acknowledged for quite some time [13]. Jaworsky et al. revealed through ultrastructural studies that inflammation plays a crucial role in AGA, as evidenced by thickened follicular sheaths, mast cell degranulation, and T cell infiltration. This inflammatory process could lead to progressive fibrosis of the perifollicular sheath and disruption of normal hair cycling, ultimately resulting in hair loss [14]. Therefore, perifollicular angiogenesis and regulation of the perifollicular immune microenvironment are expected to be therapeutic strategies for AGA hair regeneration.

Currently, the most commonly used drugs for the treatment of AGA include minoxidil, prostaglandins, aminexil, etc., this main mechanism is to promote perifollicular vascularization. Nevertheless, these drugs have limitations such as unsatisfactory treatment rates, differences in efficacy, and potential side effects [[15], [16], [17], [18]]. Autologous sources and minimally invasive approaches provide alternative treatments for AGA, such as platelet-rich plasma (PRP) therapy and the utilization of adult stem cells. The efficacy of autologous platelet-rich plasma (A-PRP) has been demonstrated in treating AGA, PRP exhibits efficacy in treating AGA by enhancing the survival of dermal papilla cells throughout the hair growth cycle via its anti-apoptotic effects [[19], [20], [21], [22]]. With the development of regenerative medicine, stem cell-based treatments have shown potential for hair regeneration and hair follicle repair as promising alternative. Stem cell therapy facilitates or orchestrates the growth, regeneration, and development of hair follicles by modulating pertinent signaling factors and pathways [23], including human follicle mesenchymal stem cells [24], mesenchymal stem cells (MSCs), and hair follicle stem cells (HFSCs) [25,26]. However, PRP therapy and stem cell therapy involve complex and cumbersome processes and are relatively expensive. Other potential AGA treatments based on promoting perifollicular angiogenesis and regulating the perifollicular immune microenvironment are worthy of further exploration. VEGF regulates blood vessel formation during embryonic development and wound healing, and is one of the most important players in maintaining vascular homeostasis in adult organisms [18,27]. Angiogenic genes are downregulated in bald scalps of AGA patients, and vascular endothelial growth factor, a critical factor in promoting microvascular permeability and angiogenesis, Goldman demonstrated that VEGF expression was significantly reduced in alopecic follicles compared to normal human hair follicles [28,29]. Ritlecitinib is an inhibitor of JAK3 and TEC kinases, blocking *γ* common chain cytokine signaling and inhibiting CD8^+^ T cells and natural killer cells (these cells known for stimulating the immune system to kill hair follicle cells). And it was demonstrated clinically significant and enduring long-term efficacy in patients with AA because of its excellent effect of promoting hair regeneration [30]. Besides, Ritlecitinib suppresses cytokine-induced phosphorylation of signal transducer and activator of transcription (STAT) mediated by JAK3-dependent cytokines, such as interleukin (IL)-2, IL-4, IL-7, IL-15, and IL-21 [31,32]. And clinical development of Ritlecitinib for vitiligo, ulcerative colitis, and Crohn's disease is ongoing in multiple countries globally [33,34]. On the other hand, current hair loss treatment research, the general method of administration is the local application or intradermal injection, or the use of roller microneedles or fractional laser to promote the penetration of locally applied drugs. These biologically active factors usually with poor transdermal permeability, and the efficacy of the drug is low due to the barrier of the stratum corneum of the skin, or the invasive administration method brings inconvenience and pain to the patient.

Recently developed microneedle (MN) technology combines the advantages of traditional intradermal injection with the convenience of transdermal drug delivery, providing a convenient, minimally invasive, and painless method of transdermal drug delivery [[35], [36], [37], [38]]. MN consist of micron-sized needles (typically less than 1 mm) arranged in an array that can penetrate the stratum corneum barrier of the skin to form micron-sized hole channels without touching pain-sensing nerves, and the loaded drug permeates into the surrounding skin tissue through the micropores to achieve minimally invasive and painless transdermal administration [39,40]. Currently reported MN transdermal delivery materials include nanoparticles [41], proteins [42], peptides [43], vaccines [44], exosomes [35], etc., and some studies have also used MN to deliver hair growth activators (such as finasteride [45], minoxidil [46], valproic acid [47], nano-preparations [5], exosomes [48], etc.) transdermal to treat hair loss, which proves that MN is feasible for AGA treatment. The types of MNs mainly include solid MNs, coated MNs, dissolving MNs, hollow MNs, and hydrogel-forming MNs [49]. Hydrogel has a three-dimensional network structure, has good biocompatibility and hydrophilicity, and can swell to several times its original volume in liquid [[50], [51], [52], [53], [54]]. The unique swelling properties of hydrogels make hydrogel forming microneedles have high drug release efficiency and can prolong the drug release period and reduce dosing frequency [55,56]. Crosslinked HA hydrogels have been used in plastic fillings [57], soft tissue fillings materials [58], and drug delivery [59] in the medical field. The carboxyl groups on HA are modified by methacrylated (HAMA) and can be crosslinked to form a hydrogel when exposed to UV light [60]. HAMA preserves HA's outstanding biocompatibility and biodegradability. By modulating the degree of methacrylation, hydrogels with customizable physical properties, such as degradation, stiffness, and pore structure, can be produced. Additionally, photo-initiated cross-linked hydrogels offer a more convenient process for microneedle preparation [61]. The use of cross-linked HAMA hydrogel as the MN tips enhances MN's mechanical strength, facilitating skin penetration with improved performance and ensuring excellent biological safety. Importantly, the utilization of crosslinked HAMA hydrogel as the MN tips can the mechanical strength of the MN, facilitating skin penetration with improved performance and ensuring excellent biological safety [62]. Furthermore, previous studies have shown that mechanical stimulation can activate the hair follicle cycle [63], and the mechanical stimulation caused by the application of MN in the skin can promote the formation of blood vessels around the hair follicle, increase the expression of hair growth-related genes, and activate hair follicle stem cells [5,64,65]. Therefore, we hypothesized that VEGF and Ritlecitinib integrated hydrogel MN could not only achieve efficient minimally invasive transdermal drug delivery, but also effectively remodel the microenvironment around hair follicles and promote angiogenesis, thereby promoting the hair regeneration therapeutic effect of AGA.

**Scheme 1:**
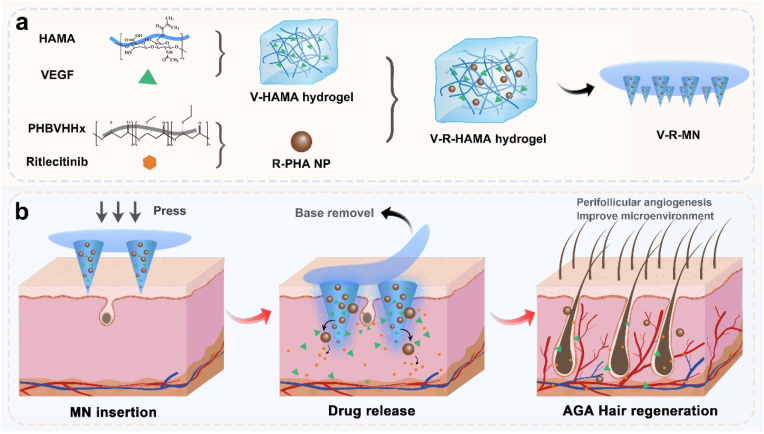
Schematic illustration of the V-R-MN integrated microneedle patch for androgenetic alopecia treatment. (a) Fabrication of V-R-MN. (b) The application of the V-R-MN patch induces appropriate mechanical stimulation, and the VEGF released by V-R-MN in the skin remodels the microvascular network around the hair follicle, and the Ritlecitinib released by the V-R-MN improves the immune microenvironment around the hair follicle, thus promoting hair follicle development and hair regrowth.

Considering these factors, a HAMA-hydrogel-forming MN (V-R-MNs) integrated with VEGF and Ritlecitinib was developed for the treatment of AGA, which can promote angiogenesis in the perifollicular microenvironment and improve the perifollicular immune microenvironment to synergistically promote hair regrowth in AGA model mouse (Scheme 1). The MN consists of swellable HAMA hydrogel tips and dissolvable polyvinyl alcohol (PVA) base, HAMA hydrogel is loaded with VEGF and hydrophobic Ritlecitinib is encapsulated by polyhydroxyalkanoate (PHA) nanoparticles (R-PHA NPs) and mixed in HAMA hydrogel. PHAs, a naturally occurring microbial biopolyester, exhibits excellent biodegradability, biocompatibility, and controllable degradation. In comparison with commonly used polyester materials like polylactic acid (PLA), which rapidly degrade, releasing lactic acid and cause non-bacterial inflammation in the implanted microenvironment, PHAs demonstrate superior long-term drug release and milder tissue stimulation, and its degradation products are safe and non-genotoxic, making it applicable in fields such as drug delivery and tissue engineering [[66], [67], [68], [69], [70], [71], [72], [73], [74], [75], [76], [77]]. The integration of HAMA hydrogel alongside PHA nanoparticles significantly bolstered the mechanical characteristics of microneedles and enhanced skin penetration efficiency. Based on the excellent mechanical strength of V-R-MN, after the microneedle is inserted into the skin, the hydrogel tip absorbs the skin interstitial fluid and swells, and the loaded VEGF and R-PHA NPs are permitted sustained release into the epidermis and dermis to reach around the hair follicles. The simultaneous release of VEGF and Ritlecitinib promotes angiogenesis in the vicinity of hair follicles, improve the immune microenvironment around hair follicles, and effectively induce the transition of the hair follicle cycle to the anagen phase. In the AGA model mouse, V-R-MN demonstrates a satisfactory hair regeneration effect characterized by high coverage, thicker diameter, and a rapid onset of the growth phase compared with the minoxidil group. Overall, V-R-HAMA microneedles realize the loading and release of dual drugs of hydrophilic VEGF and hydrophobic Ritlecitinib, and this represents a new strategy to synergistically promote AGA hair regeneration, and provides a reference for the treatment of other related diseases.

## 2 Materials and methods

### 2.1 Materials and reagents

Hyaluronic acid (HA), methacrylic anhydride, 2-hydroxy-4’-(2-hydroxyethoxy)-2-methylpropiophenone, poly (vinyl alcohol) (PVA), and Minoxidil were all purchased from Aladdin Bio-Chem Technology Co., Ltd (Shanghai, China). Poly (3-hydroxybutyric acid-*co*-3-hydroxyvaleric acid-*co*-3-hydroxyhexanoic acid trimer) (PHBVHHx or PBVHx), a novel trimer of PHA containing 90 mol% of 3-hydroxybutyrate, 3 mol% of 3-hydroxyvalerate and 7 mol% of 3-hydroxyhexanoate purchased from Bluepha Co., Ltd (Beijing, China). Testosterone was purchased from Solarbio Science & Technology Co., Ltd. (Shanghai, China). Cell Counting Kit-8 (CCK-8) was obtained from Dojindo Laboratories (Kumanoto, Japan). Calcein acetoxymethyl ester (Calcein-AM) and 3,8-diamino-5-[3-(diethylmethylammonio) propyl]-6-phenylphenanthridinium diiodide (PI), crystal violet staining solution, matrix-Gel™ (growth factor reduced) were obtained from Beyotime Biotechnology (Shanghai, China). VEGF and VEGFA ELISA Kit were purchased from ABclonal Technology Co., Ltd.

### 2.2 Materials preparation and characterization

#### 2.2.1 Preparation and characterization of methacrylate HA (HAMA)

The preparation of HAMA was referenced to the previous method [78]. Briefly, 1 g of HA was dissolved in 100 mL of deionized water and stirred for 6 h to completely dissolve it, then 5 mL of MA was added to the HA solution while controlling the pH between 8.5 and 9 by dropwise addition of NaOH solution, and the reaction mixture was stirred at 4 °C for 24 h. After that, the reaction solution was dialyzed in deionized water for 3 days, and the dialysate was changed every 4 h, and followed by freeze-drying for 3 days to obtain the HAMA product. To verify the presence of chemical groups in the samples, Fourier transform infrared spectra of the purified products (HA, MA and HAMA) between 4000 and 400 cm^−1^ were recorded on a PerkinElmer RX1 FTIR spectrometer (FTIR, Nicolet 6700, Thermo, USA).

#### 2.2.2 Preparation and characterization of HAMA hydrogels and V-HAMA hydrogels

HAMA was dissolved into 0.1 wt% photoinitiator (2-hydroxy-1-[4-(hydroxyethoxy)phenyl]-2-methyl-1-propane (I2959)) in PBS solution. UV light was irradiated for 30 s to initiate gelation and form the HAMA hydrogel. The morphology of HAMA hydrogel was observed using a scanning electron microscope (SEM).

#### 2.2.3 Preparation and characterization of PHA NPs and R-PHA NPs

To prepare PHA NPs, 100 mg of PHA powder was dissolved in 10 mL of dichloromethane. The solution was then poured into 50 mL of 1 % PVA solution, subjected to sonication and mixed for 30 s, followed by an 8-h stirring of the reaction solution. After centrifugation at 12,000 rpm and three washes with deionized water, the reaction solution was freeze-dried to obtain PHA NPs. For R-PHA NPs, 10 mg of Ritlecitinib was dissolved in 10 mL of dichloromethane along with 100 mg of PHA. This mixture was poured into 50 mL of 1 % PVA solution, sonicated, and mixed for 30 s. The reaction solution was stirred for 8 h, centrifuged at 12,000 rpm, washed three times with deionized water, and freeze-dried to obtain R-PHA NPs. The size and morphology of both PHA NPs and R-PHA NPs were analyzed using Mastersizer and SEM.

#### 2.2.4 Preparation and characterization of microneedles

MNs were fabricated using PDMS miniature round molds. The diameter of the circular MN is 17.5 mm, and each needle has a conical tip with tapered edges. The distance between the centers of every two needle tips is 700 μm, and the needle tip has a bottom diameter of 270 μm and a height of 500 μm. The MN comprises a total of 385 needlepoints, organized in a structured array.

In the experiment, 0.5 mL of 1 % HAMA solution was added to the MN mold at room temperature, and it was subjected to negative pressure defoaming and repeated 2–3 times. The processed mold was then placed in an oven at 35 °C to heat and condense the HAMA solution multiple times. Subsequently, the mold was irradiated with UV light for 1 min for photocuring. Following this, 0.4 mL of a 5 % PVA solution was added into the mold as the base of the MN. The mold was placed in an oven at 35 °C for over 12 h to ensure thorough drying. The MN was then separated from the mold to obtain the final MN product.

For visualization purposes, Rhodamine B (RB) fluorescently labeled HAMA (RB-HAMA) and Coumarin-6 (C6) labeled R-PHA NPs (C6-PHA NPs) were used for prepared fluorescent MNs. The fluorescent MNs were prepared as described above. All samples were observed using a scanning electron microscope (SEM, ZEISS Sigma 300), and the fluorescent images of the MNs were captured using laser confocal microscopy (Leica TCS SPB).

#### 2.2.5 Drug release from hydrogels

To investigate the release of VEGF from HAMA hydrogels, each hydrogel sample (Φ 6 × 4 mm^3^) was placed in a 15 mL centrifuge tube containing 10 mL of PBS (pH 6.8) and shaken in a shaker at 100 rpm at 37 °C. At pre-set time intervals, 500 μL of supernatant was taken from each tube, and then the same volume of fresh PBS was added, and the VEGFA ELISA Kit released in the supernatant was quantitatively analyzed.

To investigate the release of Ritlecitinib from R-PHA NPs, 5 mg R-PHA NPs was placed in a 15 mL centrifuge tube containing 10 mL of PBS (pH 6.8) and shaken in a shaker at 100 rpm at 37 °C. At pre-set time intervals, 500 μL of solution was taken from each tube, and centrifuged at 12,000 rpm for 30 min, then detect the amount of Ritlecitinib released in the supernatant.

### 2.3 *In vitro* cell study

#### 2.3.1 Cell viability of HDF cells

The cytocompatibility of MN extracts with HDF cells (Shanghai Cell Bank of the Chinese Academy of Sciences) was evaluated using the Cell Counting Kit-8 (CCK-8) and Live/Dead Cell Viability Kit (Invitrogen, USA). The HAMA hydrogel MN (B-MN), VEGF-loaded HAMA MN (V-MN), R-PHA NPs-loaded HAMA hydrogel MN (R-MN), and VEGF-R-PHA NPs-loaded HAMA hydrogel MN (V-R-MN) were individually immersed in complete medium for 7 days to generate distinct MN extracts for analysis. HDF cells were seeded into a 96-well plate containing 100 μL complete medium at a density of 1 × 10^5^ cells/well and incubated at 37 °C for 24 h in a 5 % CO_2_ incubator. To determine the cell viability, the medium in the well plate was removed and washed twice with PBS. Add 100 μL of medium containing 10 % (v/v) CCK-8 to each well, and continue to incubate in the incubator at 37 °C for 1 h. The absorbance at 450 nm was measured with a microplate reader.

#### 2.3.2 Cell migration of HUVECs

HUVECs (Shanghai Cell Bank of the Chinese Academy of Sciences) were seeded in a 24-well plate at a density of 5 × 10^5^ cells/well. Following 24 h incubation, the cell monolayer was scratched using a sterile pipette tip, the extract obtained by soaking the MNs in the culture medium (without fetal bovine serum) for 7 days was used for the above-mentioned cell culture. Subsequent to incubation for 24 or 48 h, the cells were fixed with 4 % paraformaldehyde and stained with crystal violet staining solution. Observation and imaging were carried out using an inverted fluorescence microscope (FM). The scratch area of the cell monolayer was quantified by measurement and calculation with Image J software (NIH, USA).

#### 2.3.3 *In vitro* angiogenesis

HUVECs were seeded on the matrix-Gel's surface of a 96-well plate at a density of 1 × 10^5^ cells/well, the extract obtained by soaking the MNs in the culture medium for 7 days was used for the above-mentioned cell culture. After a 12-h incubation, the medium was removed, and the cells were fixed with 4 % paraformaldehyde. Staining was performed with crystal violet, and tube formation was captured using a microscope. Tube lengths were measured and calculated using Image J software.

### 2.4 *In vivo* animal study

#### 2.4.1 *In vivo* MNs for AGA treatment

For the welfare of experimental animals, all animal experiments were carried out in accordance with the procedures approved by the Animal Ethics Committee of Northwest University. Healthy male C57BL/6 mice (6 weeks old, Huachuang sinoPharmaTechCo., Ltd, Jiangsu, China) were used in this study. After being housed for 1 week to acclimate, the back skin of the mice was shaved using electric hair clipper and depilated with a depilatory cream. Mice without obvious wounds on the back were selected and randomly divided into groups: Control group, Blank HAMA MN group (B-MN), VEGF-loaded HAMA MN group (V-MN), R-PHA NPs-loaded HAMA MN group (R-MN), VEGF and R-PHA NPs-loaded HAMA NN group (V-R-MN), Minoxidil group, and Ritlecitinib group. Subsequently, a slight modification was made according to the method of establishing the AGA mouse model reported previously [79]. Briefly, testosterone solutions (0.2 %, w/v) were prepared in ethanol solutions (50 %, v/v). Testosterone solution (0.1 mL/cm^2^) was topically applied to the depilated site of all mice once a day for 28 consecutive days. Testosterone solution should also be applied every day for the first 7 days before using the MN, and then take pictures every day to record the hair growth of the depilated area on the back of the mouse. On day 1, 4, 8, and 12, MNs and 5 % Minoxidil (0.1 mL/cm^2^), 5 % Ritlecitinib (0.1 mL/cm^2^) were used on the depilated sites, respectively.

#### 2.4.2 In vivo degradation of MNs

The day before the experiment began, the back skin of the mouse was shaved and depilated under isoflurane-induced anesthesia using an electric shaver and depilatory cream. The mice were thoroughly rinsed to prevent residual depilatory cream from causing skin irritation. The fluorescent MN were applied to the back skin of mouse and pressed for 5 min before removing the backing of the microneedles. On days 0, 3, 7, and 11 post-microneedle application, the fluorescent signal on the back of the mouse was scanned using an in vivo animal fluorescence imaging system (PerkinElmer, IVIS Spectrum). Mouse were sacrificed at 5 min and 11 days after microneedle application, respectively. Skin from the MN application site was harvested and embedded in tissue-embedding medium, then stored at −80 °C until sectioning using a cryostat.

#### 2.4.3 Histological evaluation of skin samples

After sacrificing the mice on the 10th day, back skin tissues were extracted and fixed in 4 % paraformaldehyde for 24 h. Subsequently, the tissues were embedded in paraffin and sliced into 5 μm thick sections. These sections underwent H&E staining, CD31 immunohistochemical staining, and Ki67, TNF-α, IL-6 immunofluorescence staining following the manufacturer's instructions. This process aimed to assess the quantity and development of hair follicles, as well as angiogenesis and microenvironment around hair follicles.

Subsequently, on the day 21, these mice were anesthetized with isoflurane and then sacrificed by cervical dislocation, back skin tissue was retrieved, fixed with 4 % paraformaldehyde for 24 h, and similarly processed by embedding in paraffin and cutting into 5 μm thick sections. These sections were then subjected to H&E staining and CD31 immunohistochemical staining, as per the manufacturer's instructions, to evaluate the condition of hair follicles and angiogenesis in the skin tissue.

### 2.5 Statistical analysis

The statistical significance of the difference between the two groups was analyzed by the two-tailed Student's *t*-test. Multiple group comparisons were performed using one-way analysis of variance. Animal experiments n = 5, all other experiments were performed 3 times. Data were obtained from at least three replicates of each data set, and all data are shown as mean ± standard deviation. In all cases, *n. s.* ≥ 0.05, *P < 0.05, **P < 0.01, ***P < 0.001, respectively.

## 3 Results and discussion

### 3.1 Fabrication and characterization of HAMA and PHA NPs

**Fig. 1:**
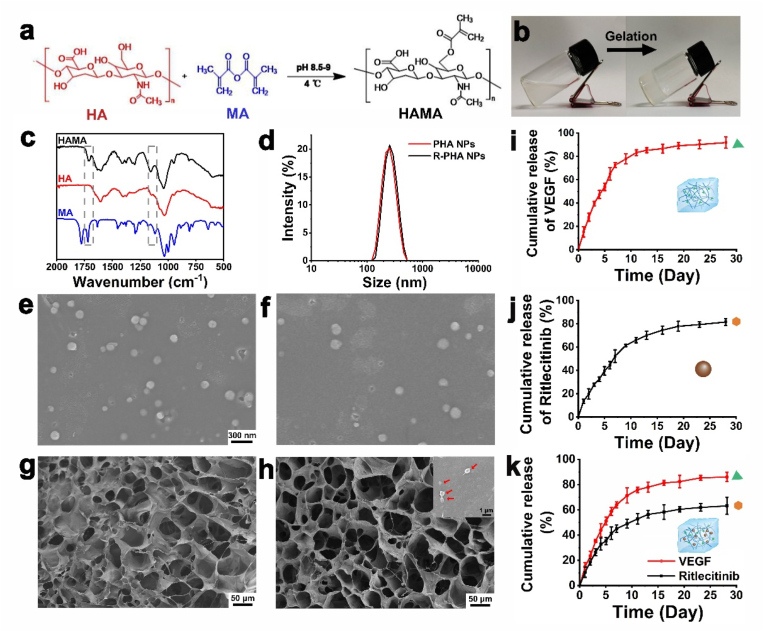
(a) Schematic illustration of the synthetic HAMA. (b) Gelation of the HAMA hydrogel. (c) FTIR spectra of the HA, MA, and HAMA. (d) Size distribution of PHA NPs and R-PHA NPs. SEM images of PHA NPs (e) and R-PHA NPs (f). SEM images of HAMA hydrogel (g) and HAMA hydrogel loaded with R-PHA NPs (h) (insets images show the R-PHA NPs in the hydrogel). (i) Release of VEGF from V- HAMA (HAMA hydrogel loaded with VEGF). (j) Release of Ritlecitinib from R-PHA (PHA NPs loaded with Ritlecitinib). (k) Release of VEGF and Ritlecitinib from V-R-HAMA (HAMA hydrogel loaded with VEGF and R-PHA).

HAMA was prepared according to the preparation process in Fig. 1a, and the HAMA solution could be gelation by irradiating 365 nm ultraviolet light for 30 s (Fig. 1b). The FTIR spectra of HAMA and HA are shown in Fig. 1c, HAMA showed new peaks at 1637 and 1700 cm^−1^, which attributed to the absorption vibration peak of C=O and the stretching vibration band of C=C in the ester bond in methacrylic anhydride [80]. Hydrophobic PHA nanoparticles were obtained by adapting a previously reported emulsification method [68], dynamic light scattering (DLS) analysis (Fig. 1d) and SEM images showed that the particle size of Ritlecitinib-loaded PHA NPs (R-PHA NPs) (Fig. 1e) did not change much from that of PHA NPs (Fig. 1f), and were uniformly distributed between 150 and 200 nm. The microstructures of HAMA and R-PHA NPs loaded HAMA hydrogels were porous with good connectivity (Fig. 1g and h). Subsequently, the release of VEGF in HAMA hydrogel and Ritlecitinib in R-PHA NPs were explored respectively, after 28 days, the release of VEGF in the HAMA hydrogel and Ritlecitinib in PHA could be released by more than 80 % (Fig. 1i and j). On the other hand, the release of VEGF and Ritlecitinib were detected on the V-R-HAMA hydrogel, the release rate of VEGF reached 92 % after 28 days, and the release rate of Ritlecitinib was 69 % (F 1 k). Notably, which was slightly lower than the individual release rates from PHA NPs alone, indicating a potential protective effect of the hydrogel network on the nanoparticles. Furthermore, the release of VEGF and Ritlecitinib in the V-R-HAMA hydrogel exhibited an initial burst release within the first 7 days, followed by a stable sustained release. This dynamic release pattern is advantageous for long-term sustained drug release into the skin post-insertion of hydrogel-forming MN. It ensures prolonged drug presence in the body, so that the drug can be maintained in the body for a longer time, have a longer acting time, and achieve better hair regeneration effects. This intricate drug delivery system showcases the potential of HAMA hydrogels forming MN loaded with PHA nanoparticles, providing a platform for optimized and sustained drug release for enhanced therapeutic effects in skin applications.

### 3.2 Fabrication and characterization of the V-r-mns

**Fig. 2:**
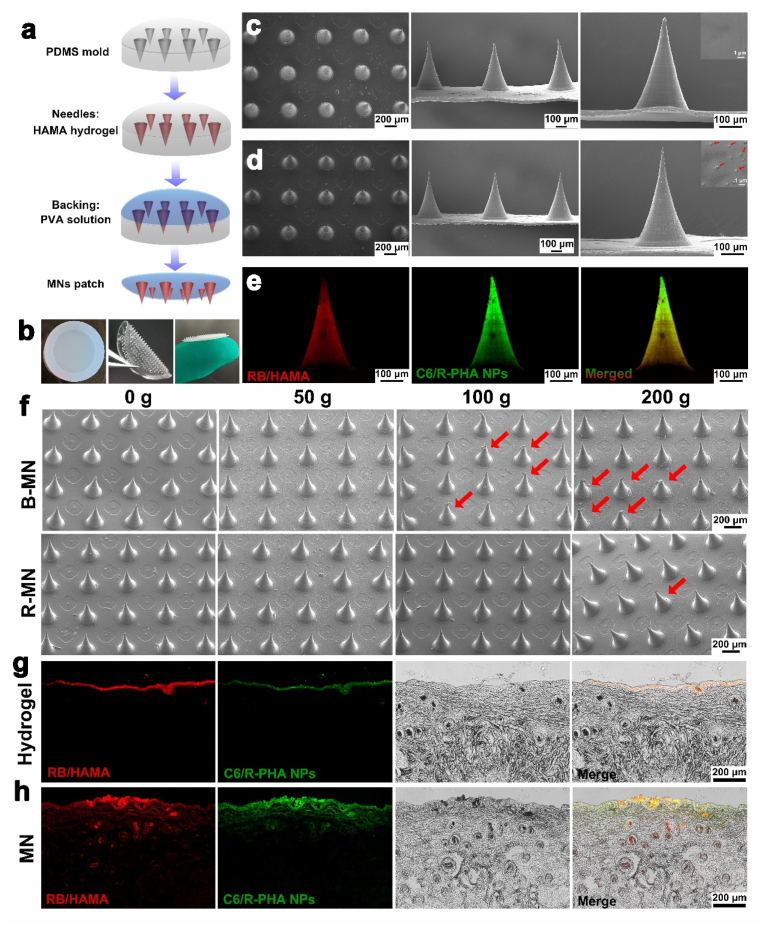
(a) Schematic diagram of the fabrication process of HAMA MNs. (b) Photos of HAMA hydrogel in PDMS mold and HAMA MNs. SEM images of the blank HAMA hydrogel-forming microneedle (B-MN) patch (c), R-PHA NPs loaded HAMA hydrogel-forming microneedle (R-MN) patch (d). (e) Fluorescence microscopy images of the R-PHA NPs loaded HAMA MNs (containing RB-labeled HAMA, and containing Coumarin 6-labeled R-PHA NPs). (f) Morphological changes of B-MN and P-MN after loading different mass weights (0, 50, 100 and 200 g) for 5 min. Representative fluorescence images of skin sections 24 h after topical application of fluorescence HAMA hydrogel (g) and fluorescence MN (h), containing Rhodamine B-labeled HAMA loaded containing Coumarin 6-labeled R-PHA NPs.

To achieve the transdermal delivery of VEGF and R-PHA NPs, we employed HAMA hydrogel microneedles for the simultaneous encapsulation of VEGF and R-PHA NPs, enabling penetration of the skin barrier for effective drug delivery. The fabrication process of HAMA MN is illustrated in Fig. 2a. In this process, the HAMA solution, containing VEGF and R-PHA NPs, was filled into the MN mold and exposed to UV light for 30 s for gelation. Subsequently, PVA solution was added as the backing material, and the fully dried HAMA hydrogel MN was separated from the mold. Fig. 2b presents a digital photograph of the MN mold and the prepared HAMA MNs the MN has a certain flexibility, and its diameter is 17 mm. SEM images of the manufactured MN (Fig. 2c) reveal a needle base diameter of about 270 μm, a needle tip height of approximately 500 μm, and a distance of 700 μm between needles, with a total of 385 needles in a MN. Fig. 2d displays SEM images of the R-MN, some PHA NPs ware founded on the needle surface indicated by red arrows, and without altering the overall morphology and structure of the MNs. To visualize drug loading in the MNs, R-PHA NPs were labeled with Coumarin-6 (green fluorescence) and HAMA hydrogel with Rhodamine B (red fluorescence). The confocal fluorescence microscope images (Fig. 2e) depict even distribution of R-PHA NPs on the MNs needle.

A weights-bearing experiment was conducted to assess the hardness of the needles, applying weights of 50 g, 100 g, and 200 g to B-MN and R-MN for 5 min. Under 50 g of weight, the shape of the needle tips of B-MNs and R-MNs remained almost unchanged as shown in Fig. 2f. At 100 g, a few MN tips of B-MN broke at the edges, while almost all tips of R-MN remained upright. Under 200 g, about 12 % of B-MN tips were broken and deformed, while the number of breakages of R-MN was about 3.5 %, which was much less than that of B-MN (Figs. S1 and S2). This indicates that B-MN possessed excellent mechanical strength and hardness, and the incorporation of PHA NPs further enhanced the mechanical strength and hardness of the MN, enabling it to withstand a certain degree of gravitational pressure. This enhancement in hardness is attributed to the incorporation of nanoparticles, which reinforces the mechanical strength of the hydrogel, facilitating smooth penetration into the stratum corneum for minimally invasive transdermal drug delivery. Similar studies have also indicated that the addition of nanoparticles can significantly enhance the mechanical strength of microneedles. To demonstrate the transdermal drug delivery effect of MNs, Rhodamine B-labeled HAMA hydrogel (RB/HAMA), mixed with Coumarin 6-labeled R-PHA NPs (C6/R-PHA), was applied to the skin surface. After 24 h, fluorescence was solely observed on the skin's surface without penetrating into the skin (Fig. 2g). The skin was then subjected to a 24-h treatment with the fluorescent MN, prepared using the aforementioned hydrogels. Observations revealed widespread distribution of both red and green fluorescence on the skin (Fig. 2h), indicating that the MN could successfully penetrate the stratum corneum barrier and deliver the encapsulated drug directly to the hair follicles situated 200–300 μm below the skin. Similar studies also indicate that MNs possess appropriate morphological structures, enabling them to directly overcome the stratum corneum barrier, puncturing the skin and transporting the loaded drug to the follicular regions [5,81]. These results affirm the excellent morphological structure and suitable mechanical properties of the PHA NPs with hydrogel forming MNs, enabling successful penetration of the skin's stratum corneum and effective delivery of loaded drugs to the hair follicles.

### 3.3 Biocompatibility and influence on cell migration and angiogenesis of MN

**Fig. 3:**
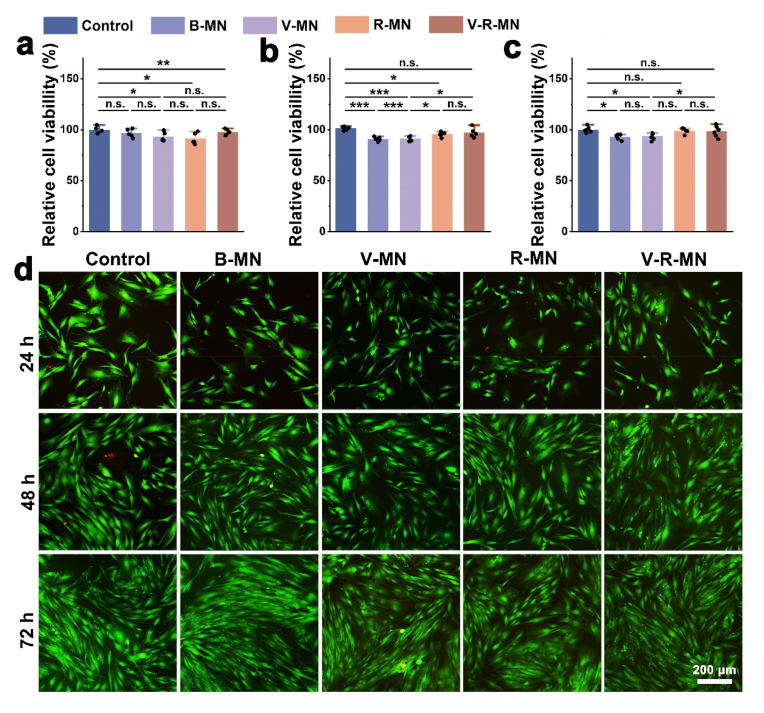
Relative viabilities of HDF cells co-incubated with diverse MN patchs extracts for 24 h (a), 48 h (b), 72 h (c), respectively. d) Calcein-AM (live cells, green)/PI (dead cells, red) double staining of HDF cells subjected to different treatments with MN patchs extracts for 24 h, 48 h, and 72 h, respectively. B-MN: Blank HAMA MN, V-MN: VEGF loaded HAMA MN, R-MN: R-PHA NPs loaded HAMA MN, V-R-MN: VEGF and R-PHA NPs loaded HAMA MN. *n. s.* ≥ 0.05, *p < 0.05, **p < 0.01, ***p < 0.001 (n = 5).

**Fig. 4:**
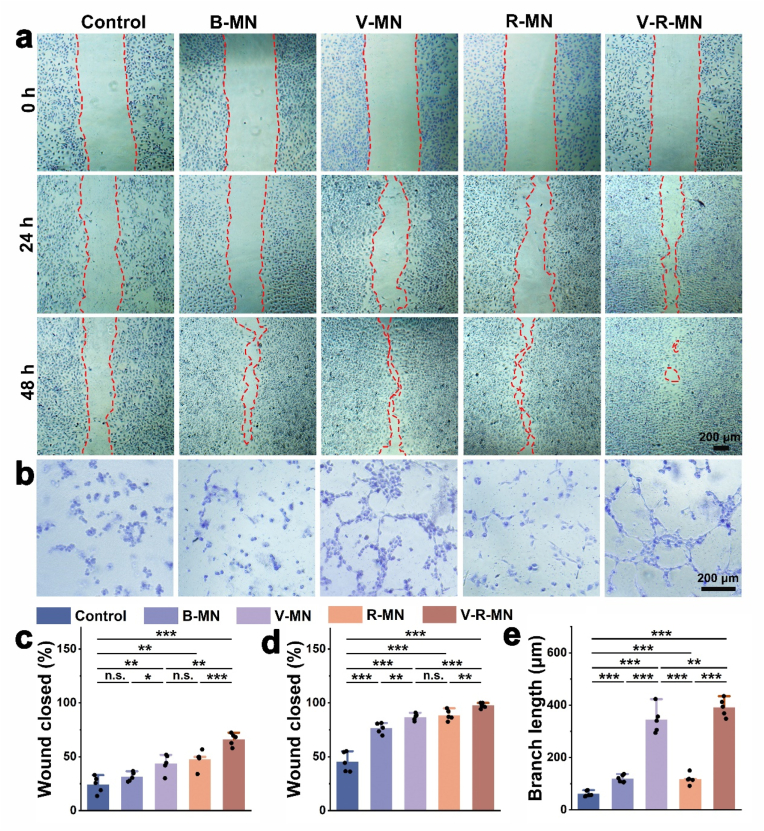
(a) Effects of MN patchs extracts on the migration of HUVECs from at 24 h and 48 h. (b) Representative micrographs of tube formation of HUVECs in the presence of MN patchs extracts for 12 h. Cell migration rate of HUVECs after 24 h (c) and 48 h (d) incubation. (e) Quantification of the mesh-like tube length in MN patchs extracts-treated HUVECs. *n. s.* ≥ 0.05, *p < 0.05, **p < 0.01, ***p < 0.001 (n = 5).

An ideal drug delivery platform should have excellent biocompatibility. To further verify the biocompatibility of the MNs, the cell viability of different MNs and cells co-cultured for different time was determined by CCK-8 assay and Calcein-AM/PI staining. Compared with the control group (common medium), each experimental group showed higher biocompatibility after co-cultivation with HDF cells for 24, 48, and 72 h (Fig. 3a–c). In addition, the Calcein-AM/PI staining results of each component of the system under the same conditions (Fig. 3b) also demonstrated consistent experimental results. Compared with widely studied biodegradable polymers in biomedical applications, such as polylactic acid (PLA) and polylactic acid-polyethylene glycol copolymer (PLGA), PHA exhibits higher biocompatibility, its surface properties and chemical structure help reduce adverse reactions of organisms to the material, making it more compatible with living tissues and organisms [72,[82], [83], [84], [85]]. In addition, PHA as hydrophobic drug carriers for controlled release offer advantages due to their tunable properties, high biocompatibility, and biodegradability, providing an effective and sustainable solution for drug delivery. The adjustability of PHA's physical and chemical characteristics allows tailored drug release, while its biocompatibility and biodegradability minimize adverse effects, making it a promising material for controlled release of hydrophobic drugs [86,87]. As a key player in angiogenesis, VEGF is attenuated in the perifollicular environment of patients with androgenetic alopecia, the newly formed blood vessels around the hair follicles can provide the hair follicles with nutrients and oxygen needed for development and growth, thereby promoting hair regeneration [28,29]. Therefore, the migration, proliferation, and tube formation of HUVECs are essential steps in the angiogenesis process, affecting the effect of hair regeneration. As shown in Fig. 4a, after 24 h, the migration rate of the V-R-MN group was higher (68.27 %) (Fig. 4c), and after 48 h, the migration rate of the V-R-MN group reached 96.20 % (Fig. 4d). Meanwhile, as shown in Fig. 4b and e, the V-MN and V-R-MN groups showed more visible tube-like tube structures and more tube numbers in the results of promoting HUVEC tube formation experiments. According to previous research results, critical cytokines of angiogenesis such as VEGF, FGF2, and HGF can induce multiple angiogenesis genes and are crucial in angiogenesis [88,89]. Therefore, these results indicate that V-R-MN can effectively release VEGF and has an excellent ability to promote cell migration and angiogenesis, and angiogenesis is beneficial to promote the development of hair follicle cells and improve the hair follicle microenvironment.

### 3.4 Hair regrowth evaluation

**Fig. 5:**
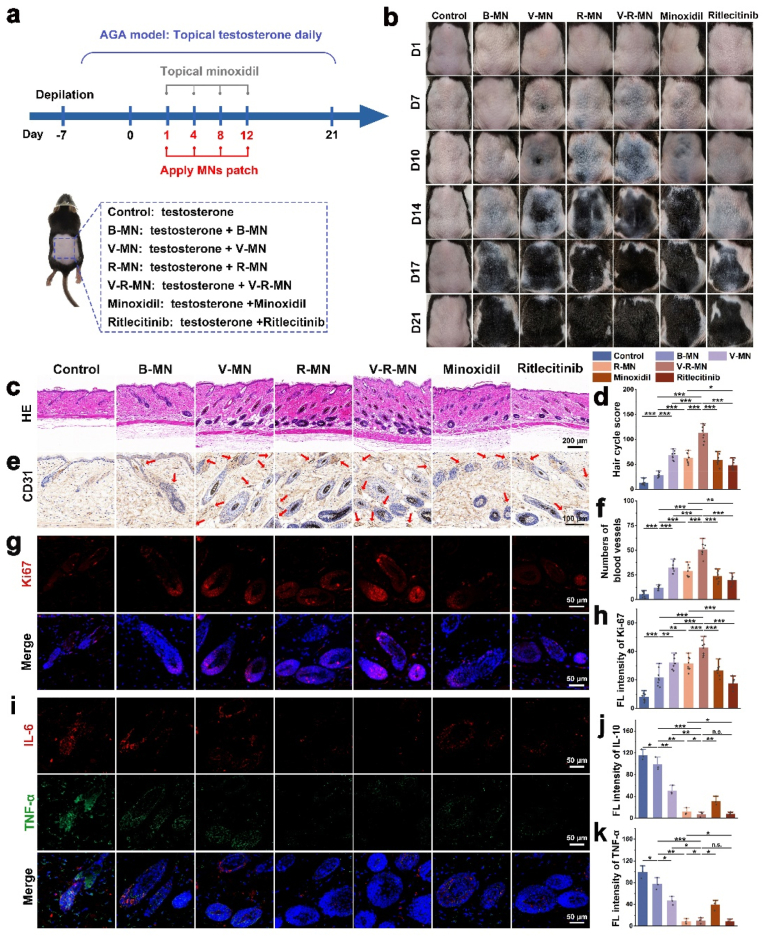
(a) Schematic diagram of AGA mouse model establishment with topical application of testosterone solution daily for 28 consecutive days and the treatment strategies of each group of mice in the established model. In the control group, the depilated area was only treated with testosterone solution, and in the other experimental groups, MNs or Minoxidil were applied for 4 times with an interval of 3 days each time. (b) Representative photographs of mouse hair regrowth status in different groups. (c) H&E staining of the treated skin for different groups at day 10. (d) Hair cycle score at day 10 (n = 5). (e) Representative images of Immunohistochemistry staining of CD31 on depilated skin of different groups to investigate perifollicular angiogenesis on day 10 (red arrows point to blood vessels). (f) Quantification of numbers of blood vessels in each field (n = 5). (g) Representative orescence staining of Ki67 on depilated skin of different groups on day 10 (red: Ki67, blue: DAPI). (h) Quantitative analysis of Ki67 positive cells at day 10 in each field (n = 7). (i) Representative images of immunofluorescence staining of TNF-α and IL-6 on depilated skin of different groups on day 10 (red: IL-6, green: TNF-α, blue: DAPI). Quantitative analysis of IL-6 (j) and TNF-α (k) positive cells at day 10 in each field (n = 3).

**Fig. 6:**
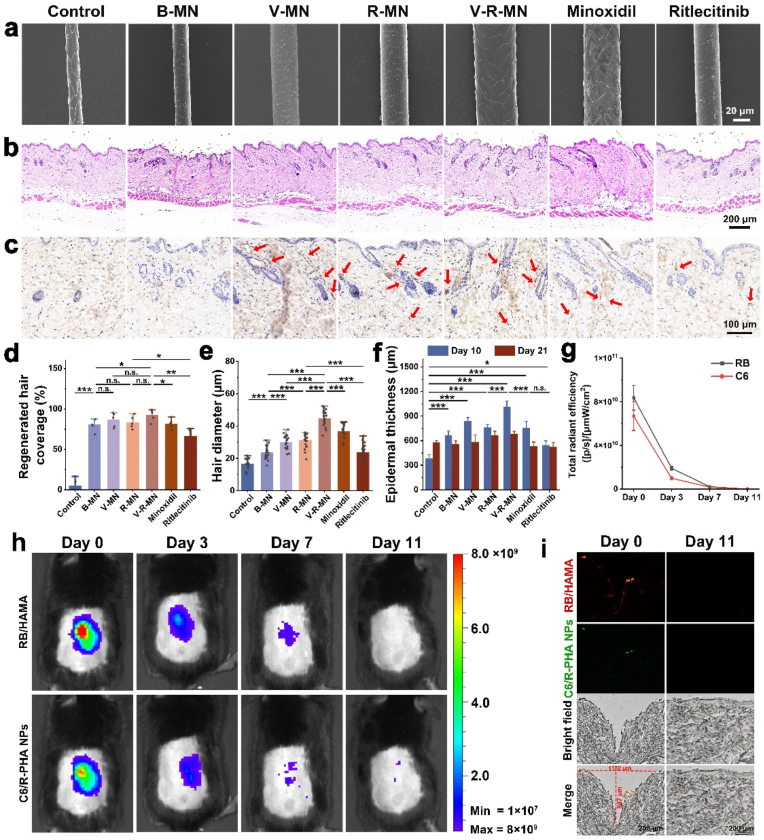
(a) SEM images of regenerated hair of different groups at day 21. (b) H&E staining of the treated skin of different groups at day 21. (c) Representative images of Immunohistochemistry staining of CD31 on depilated skin of different groups to investigate perifollicular angiogenesis on day 21 (red arrows point to blood vessels). (d) Diameter of regenerated hair of different groups at day 21 (n = 5). (e) The ratio of the regenerated hair coverage area to the depilated area in each group on day 21 (n = 20). (f) Skin thickness on day 10 and day 21 (n = 7). (g) Quantitative analysis of TNF-α and IL-6 positive cells at day 10 in each field (n = 3). (h) IVIS images of the MN site of mice on the day 11 after application of fluorescent MN (containing Rhodamine B-labeled HAMA, containing Coumarin 6-labeled R-PHA NPs). (i) Representative images of the skin sections after the fluorescent MN were inserted into the mouse skin on day 0 (5 min) and day 11. *n. s.* ≥ 0.05, *p < 0.05, **p < 0.01, ***p < 0.001.

To assess the efficacy of V-R-MN in promoting hair regeneration, a testosterone-induced androgenetic alopecia (AGA) mouse model was established, and various treatment regimens were implemented as illustrated in Fig. 5a. The control group received no treatment except for daily topical application of testosterone. Different MN treatment groups applied various MN formulations on days 1, 4, 8, and 12. The positive control group received topical application of 3 % Minoxidil, the final group was topically treated with Ritlecitinib. The hair follicle growth cycle involves anagen, catagen, and telogen phase, Skin pigmentation occurs as the hair transitions from catagen to anagen due to melanin production in the hair follicles, so skin pigmentation can be considered a visual indicator of the transition from telogen to anagen [90,91]. Significantly, the depilated areas of the control group remained pink until day 21 with little regrowth of hair, demonstrating the successful establishment of testosterone-induced AGA, in contrast, treatment areas of V-MN, R-MN, and V-R-MN began to show melanin deposition as early as day 7, with the blank MN group (B-MN) and Minoxidil group showing melanin deposition from day 10 onwards (Fig. 5b). All MNs and Minoxidil treatment groups exhibited significant hair regrowth in the treatment areas. Specifically, the V-R-MN group achieved superior hair coverage (Fig. 6d) and larger diameter of regenerated hair at day 21 (Fig. 6a–e) compared to the Minoxidil group. The hair regeneration effect of V-R-MN surpassed that of V-MN and R-MN monotherapy groups, confirming the positive influence of VEGF and Ritlecitinib on hair follicle development. Interestingly, even the blank MN (B-MN) group induced some hair regeneration, which may be caused by mechanical stimulation of MNs [5,63,92], while the hair growth rate and quality in the B-MN group were lower than in other MN groups (Fig. S6a, S6d and S6e), an observable effect on hair regeneration was evident. On day 10, high-density enlarged hair follicles were observed in the subcutaneous tissue of the treated areas for all groups except the control, with the V-R-MN group exhibiting significantly more anagen-phase hair follicles than other groups (Fig. 5c and d), suggesting that VEGF and Ritlecitinib can activate telogen hair follicles in the hair loss area. Additionally, on day 10, investigation into perifollicular angiogenesis revealed increased blood vessel formation in the alopecia area treated with blank MNs, likely due to mechanical stimulation from MN application. Compared to the control and Minoxidil groups, the V-R-MN group showed the highest number of blood vessels (Fig. 5e and f, Fig. S3). In addition, the number of blood vessels in each group also maintained this trend at 21 days (Fig. 6c, Fig. S4). The R-MN group exhibited more blood vessels than the Minoxidil group, possibly due to the enhanced effect of Ritlecitinib on the immune microenvironment around the hair follicle. Furthermore, Ki67 expression levels, a marker of cell proliferation, were higher in the V-MN, R-MN, and V-R-MN groups than in the Minoxidil group on day 10, with the V-R-MN group showing the highest Ki67 expression (Fig. 5g and h). These results indicate that Ritlecitinib may promote the proliferation of hair follicle growth-related cells.

Local inflammation in follicular microenvironment is the key risk factor for AGA, including 5-dihydrotestosterone (DHT), inflammation, and oxidative stress, so studying anti-inflammatory markers/drugs is of great significance in the treatment of this disease, other biomarkers for hair loss disorders are *C*-reactive protein, fibrinogen, tumor necrosis factor-α (TNF-α), and interleukin-6 (IL-6) [93,94]. And dermal papilla cells (DPCs) respond to DHT by producing IL-6 and transforming growth factor (TGF-2), leading to hair development inhibition and premature onset of the catagen stage in AGA patients [[95], [96], [97], [98]]. Therefore, the expression levels of TNF-α and IL-6 in the skin at the site of MN application on day 10 of starting treatment were investigated to evaluate the hair follicle immune microenvironment in AGA mice. In Fig. 5i, j and 5k, lower TNF-α and IL-6 expression was observed in the R-MN, V-R-MN, and Ritlecitinib groups compared to the Control group. Additionally, the V-MN group also exhibited lower levels compared to the Control group, possibly due to enhanced hair follicle microenvironment resulting from angiogenesis. This result fully demonstrates that Ritlecitinib positively influences the hair follicle immune microenvironment in AGA mice, thereby promoting hair regeneration.

To evaluate the in vivo biocompatibility of MNs, the thickness of the skin of each experimental group was measured on day 10 and day 21. The results showed that skin thickness was significantly higher in all groups than in the model group on day 10, decreasing after day 21 (Fig. 6f). This fluctuation may be attributed to the variation in skin thickness associated with the hair follicle cycle. As the hair follicles transition from the resting phase to the anagen phase, the high-density enlarged hair follicles increase the thickness of the skin, and then the hair follicles enter the mid-to-late growth and recession phases and the hair follicles shrink, resulting in a reduction in skin thickness. This indirectly illustrates the hair regeneration effects of V-MN, R-MN, and V-R-MN. Additionally, skin tissues treated with MNs in each group showed no apparent damage on days 10 and 21 (Figs. 5c and 6b), respectively, confirming the high biocompatibility of V-R-MNs. This suggests that the MN-based delivery system is well-tolerated by the skin, and underscore the potential of V-R-MNs as a safe and efficient treatment for AGA, further supporting its potential for clinical applications.

### 3.5 In vivo degradation of MN

The degradation of microneedles in mice was explored by applying fluorescent microneedles to the back skin and removing the backing. Changes in fluorescence signal were continuously observed using a small animal in vivo imaging system. Over time, the signal gradually weakened until nearly disappearing after 11 days (Fig. 6i–g). In addition, no obvious fluorescence signal was found in the main internal organs of the mice on Day 11 (Fig. S5), which confirmed the safe degradation of the MN in the mice. Microscopic examination of mouse skin tissue sections after 5 min of microneedle application revealed channels formed by microneedle insertion, with red and green fluorescent signals along the edges (Fig. 6i). The triangular-shaped channel is consistent with the conical structure of the microneedle tip. The channel is 1182 μm wide and 947 μm high, which is much larger than the size of the microneedle tip itself (needle base diameter 270 μm, needle height 500 μm). This result fully demonstrates that the hydrogel MN have appropriate mechanical strength to penetrate the skin, and instantly absorb body fluids and swell after entering the skin, which is more conducive to the passage of drug molecules loaded inside the microneedles, the drug can effectively penetrate the stratum corneum and its transdermal permeability is greatly increased. By the day 11, fluorescence signals on skin tissue sections nearly vanished, and the skin surface returned to a flat state, indicating reversible and easily reparable minimally invasive damage caused by MN application. Overall, V-R-MN hydrogel microneedles exhibit sufficient mechanical strength and biological safety, facilitating minimally invasive transdermal drug delivery.

Previous studies have confirmed that mechanical stretching, under specific tension and duration conditions, activates hair stem cells and promotes hair growth [5,63,81,99]. Our experimental results also confirmed that mechanical stimulation of blank MN was beneficial for the growth of hair follicles. Notably, mice treated with locally applied Ritlecitinib and Minoxidil exhibited significantly poorer hair regeneration compared to various MN groups, with delayed onset, indicating lower transdermal efficiency of topically applied drugs. This emphasizes the limitations of poor absorption and lower transdermal efficiency of topically applied drugs. It is noteworthy that the V-R-MN treatment group exhibited a faster hair regeneration rate and superior quality compared to monotherapy (V-MN and R-MN) and the Ritlecitinib and Minoxidil group. This emphasizes the direct delivery of drugs to the target area through microneedles, minimizing drug wastage on the body surface, and demonstrates the role of microneedles in enhancing transdermal drug delivery in a minimally invasive manner. These results collectively demonstrate the effectiveness of V-R-MNs in promoting hair regeneration, with significant implications for improving treatment outcomes in AGA. Further research is warranted to explore the underlying mechanisms and optimize the MN matrix materials and delivery system for enhanced clinical efficacy.

## 4 Conclusion

In this study, a hydrogel-forming MN V-R-MN was developed incorporating VEGF and R-PHA NPs. The addition of R-PHA NPs enhances the mechanical hardness of the hydrogel MN, facilitating its penetration of the stratum corneum for minimally invasive transdermal drug delivery. This MN system with excellent skin penetration ability, significantly enhances the release of VEGF and R-PHA NPs in the skin. The synergistic effect of mechanical stimulation from the V-R-MN and the promotion of angiogenesis and perifollicular immune microenvironment remodeling resulted in a more satisfactory hair regeneration effect in AGA mice compared to the commercial drug Minoxidil. Importantly, our study reveals the limitations of topically applied Minoxidil and Ritlecitinib, demonstrating lower transdermal efficiency and delayed onset of hair regrowth. This emphasizes the potential of MNs to enhance drug delivery in a minimally invasive and painless manner. The observed high biocompatibility of V-R-MNs with skin tissues further supports the safety and feasibility of this delivery method. While our study provides valuable insights, continued research is necessary to elucidate the underlying mechanisms and optimize the MN system for broader clinical applications. In summary, the results indicate that V-R-MNs hold promise as an innovative and effective approach for the treatment of AGA, offering potential benefits in terms of both therapeutic outcomes and patient comfort.

## 5 Ethics approval and consent to participate

This study and included experimental procedures were approved by resolution of Northwest University Committee on experimental animal management and ethics (Approval No. NWU-AWC-20211202 M). All animal housing and experiments were conducted in strict accordance with the institutional guidelines for care and use of laboratory animals.

## Conflicts of interest

The authors declare no conflicts of interest.

## CRediT authorship contribution statement

**Yan-Wen Ding:** Writing – review & editing, Writing – original draft, Methodology, Investigation, Data curation, Conceptualization. **Yang Li:** Investigation, Data curation. **Zhi-Wei Zhang:** Validation, Formal analysis. **Jin-Wei Dao:** Funding acquisition. **Dai-Xu Wei:** Writing – review & editing, Funding acquisition.

## Declaration of competing interest

The authors declare no competing financial interests or personal relationships that could have appeared to influence the work reported in this paper.
