# Supplementary material for: Hydrogel forming microneedles loaded with VEGF and Ritlecitinib/polyhydroxyalkanoates nanoparticles for mini-invasive androgenetic alopecia treatment
Source: Bioact Mater. 2024 Apr 23;38:95–108. doi: 10.1016/j.bioactmat.2024.04.020 (PMC11061199; doi:10.1016/j.bioactmat.2024.04.020)
Supplement: Multimedia component 1 [file mmc1.docx]

**Supplementary Information**

**Hydrogel forming Microneedles loaded with VEGF and Ritlecitinib/Polyhydroxyalkanoates Nanoparticles for Mini-invasive** **Androgenetic Alopecia Treatment**

Yan-Wen Ding^1^, Yang Li^1^, Zhi-Wei Zhang^1^, Jin-Wei Dao^1,4^, Dai-Xu Wei^1,2,3,5^ *

^1^Key Laboratory of Resource Biology and Biotechnology in Western China, Ministry of Education, School of Medicine, Department of Life Sciences and Medicine, Northwest University, Xi'an, 710069, China

^2^Zigong Affiliated Hospital of Southwest Medical University, Zigong Psychiatric;

^3^School of Clinical Medicine, Qujing Medical College, Qujing, 655000, China;

^4^Dehong Biomedical Engineering Research Center, Dehong Teachers’ College, Dehong, Yunnan Province, China;

^5^Shaanxi Key Laboratory for Carbon Neutral Technology, Xi'an, 710069, China.

* Corresponding author: Dai-Xu Wei*

E-mail address: weidaixu@nwu.edu.cn (D. X. Wei)


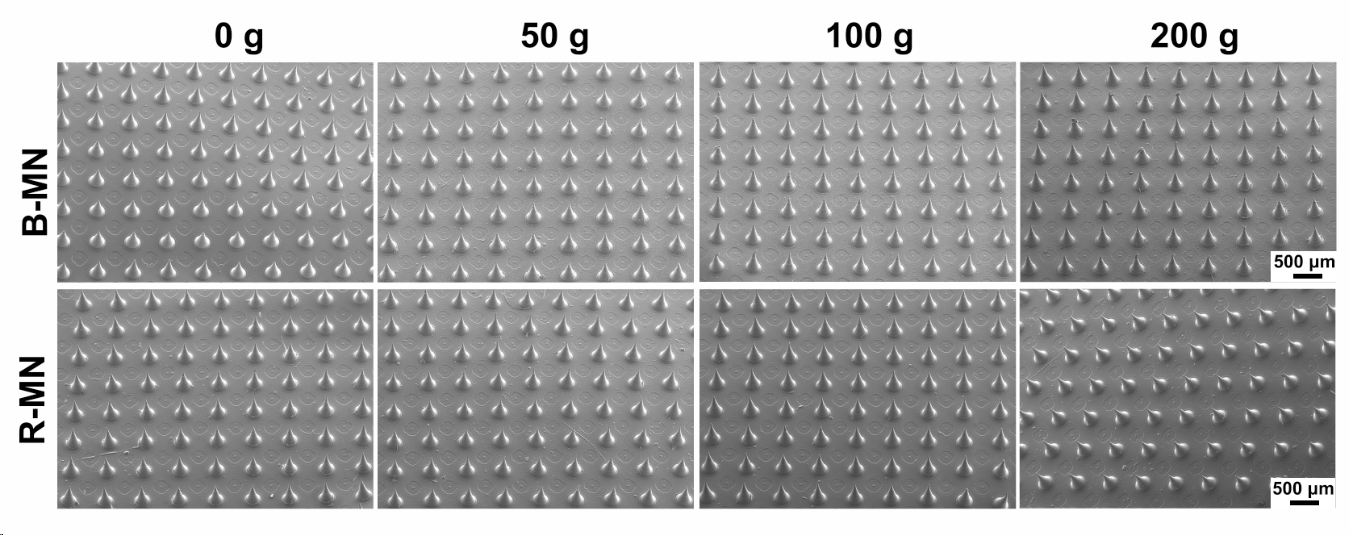


**Fig. S1.** Morphological changes of B-MN and P-MN after loading different mass weights (0, 50, 100 and 200 g) for 5 min.

**
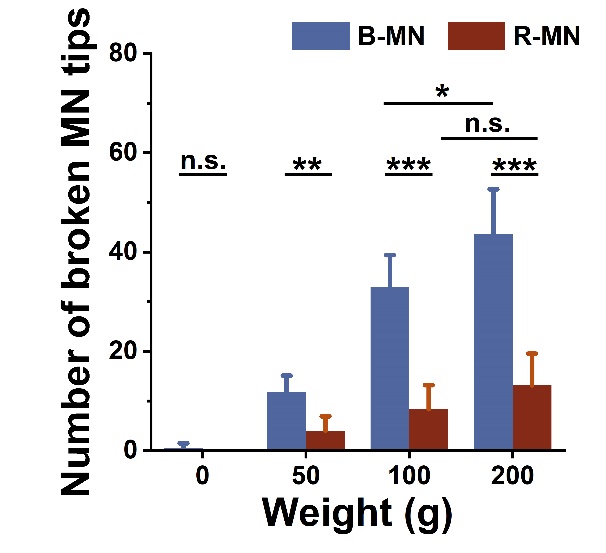
**

**Fig. S2.** The number of broken needle tips of B-MN and R-MN after loading with different masses (0, 50, 100 and 200 g) for 5 minutes. (n = 5)

**
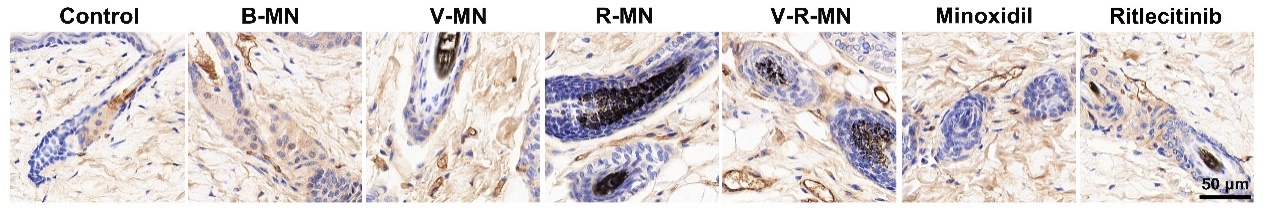
**

**Fig. S3.** Representative images of Immunohistochemistry staining of CD31 on depilated skin of different groups to investigate perifollicular angiogenesis on day 10.

**
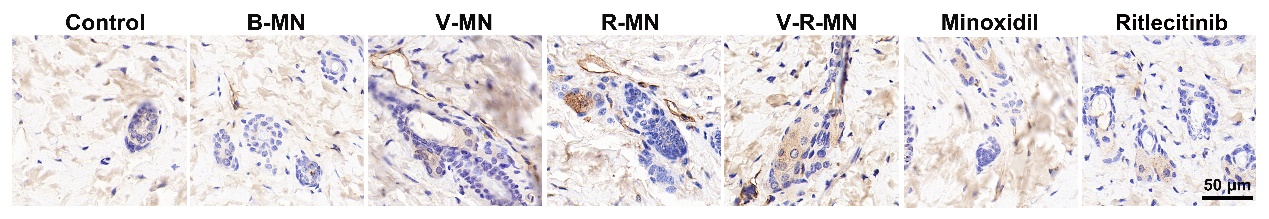
**

**Fig. S4**. Representative images of Immunohistochemistry staining of CD31 on depilated skin of different groups to investigate perifollicular angiogenesis on day 21.


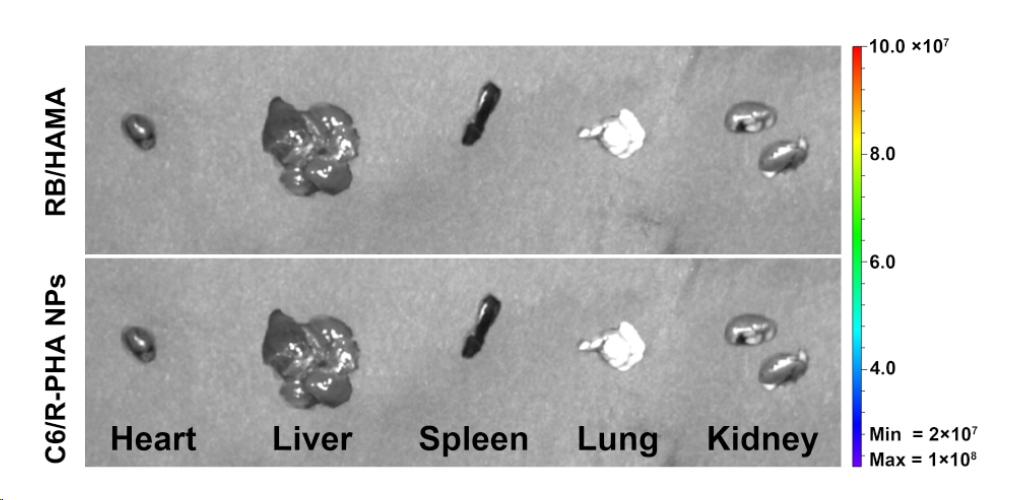


**Fig. S5**. IVIS images of the main internal organs of mice on day 11 after application of fluorescent MN (containing rhodamine b-labeled HAMA, containing coumarin 6-labeled R-PHA NPs).
